# A prediction tool for malnutrition and sarcopenia in patients with gastroenteropancreatic neuroendocrine neoplasms: results from NUTRIGETNE (GETNE-S2109) study

**DOI:** 10.3389/fnut.2026.1789458

**Published:** 2026-05-26

**Authors:** Maria Argente-Pla, Miguel Antonio Sampedro-Nuñez, Rocio Garcia-Carbonero, Ana Custodio, Jorge Hernando, Lorena Suarez, Lorena Hernandez-Rienda, Inmaculada Peiró, Vicente Alonso, Raquel Serrano, Nuria Valdés, Marcos Melian, Beatriz Febrero, Josefina Biarnes, José Ángel Díaz-Pérez, Javier Molina-Cerrillo, Carlos Lopez, Miguel Ángel Martínez Olmos, Juan Francisco Merino-Torres, Jaume Capdevila, Maribel del Olmo-García, NUTRIGETNE GROUP

**Affiliations:** 1 Joint Research Unit on Endocrinology, Nutrition and Clinical Dietetics, University of Valencia-Health Research Institute La Fe, Valencia, Spain; 2 Department of Endocrinology and Nutrition, University and Polytechnic Hospital La Fe, Valencia, Spain; 3 Department of Medicine, Faculty of Medicine, University of Valencia, Valencia, Spain; 4 Endocrinology and Nutrition Department, Hospital de La Princesa, Madrid, Spain; 5 Medical Oncology Department, Hospital Universitario 12 de Octubre, Imas12, Facultad de Medicina, UCM, Madrid, Spain; 6 Medical Oncology Department, Hospital Universitario La Paz, Instituto de Investigación Biomédica Hospital Universitario La Paz (IdiPAZ), Madrid, Spain; 7 Medical Oncology Department, Vall Hebron University Hospital, Vall Hebron Institute of Oncology (VHIO), Barcelona, Spain; 8 Department of Endocrinology and Nutrition, Hospital Universitario Central de Asturias, Instituto de Investigación Sanitaria del Principado de Asturias (ISPA), Oviedo, Spain; 9 Clinical Nutrition Unit, Institut Català d'Oncologia (ICO), l'Hospitalet de Llobregat, l'Hospitalet de Llobregat, Spain; 10 Bellvitge Biomedical Research Institute (IDIBELL), Barcelona, Spain; 11 Medical Oncology Department, Instituto Aragones de Investigacion Sanitaria, Hospital Universitario Miguel Servet, Zaragoza, Spain; 12 Medical Oncology Department, Hospital Universitario Reina Sofía, Instituto Maimonides de Investigación Biomedica (IMIBIC), Córdoba, Spain; 13 Endocrinology and Nutrition Department, University Hospital Cruces, Bilbao, Biobizkaia, CIBERDEM/CIBERER, EndoERN, UPV/EHU, Barakaldo, Spain; 14 Medical Oncology Department, Instituto Valenciano de Oncología (IVO), Valencia, Spain; 15 Department of Endocrine Surgery, General Surgery Service, Virgen de la Arrixaca University Hospital, Murcia, Spain; 16 Endocrinology and Nutrition Department, Hospital Universitari de Girona Dr. Josep Trueta, Girona, Spain; 17 Endocrinology and Nutrition Department, Hospital Universitario Clínico San Carlos, Madrid, Spain; 18 Medical Oncology Department, Hospital Universitario Ramón y Cajal, Madrid, Spain; 19 Medical Oncology Department, Hospital Universitario Marqués de Valdecilla, IDIVAL, UNICAN, Santander, Spain; 20 Endocrinology and Nutrition Department, Complejo Hospitalario Universitario de Santiago, Santiago de Compostela (A Coruña), Spain; 21 Molecular Endocrinology Group-Health Research Institute of Santiago de Compostela-IDIS, Santiago de Compostela (A Coruña), Spain; 22 CIBER de Fisiopatología de La Obesidad y Nutrición (CIBEROBN), Instituto de Salud Carlos III, Madrid, Spain

**Keywords:** gastroenteropancreatic, malnutrition, neuroendocrine neoplasms, predictive tools, prognostic factors, sarcopenia

## Abstract

**Introduction:**

Malnutrition and sarcopenia are prevalent among patients with gastroenteropancreatic neuroendocrine neoplasms (GEP-NENs), necessitating simple assessment tools to identify potentially malnourished patients. After describing the nutritional status of those receiving anticancer treatment in Spain, the development of a nutrition screening tool for GEP-NENs was proposed as an exploratory objective of the NUTRIGETNE study.

**Methods:**

Malnutrition was defined by Global Leadership Initiative on Malnutrition (GLIM) criteria. Sarcopenia by European Working Group on Sarcopenia in Older People 2 (EWGSOP2) criteria. Patients were randomized 4:1 into training and validation sets to build a least absolute shrinkage and selection operator (LASSO) model. The model was corrected by sex, age, and body mass index. Predictors selected according to the area under the curve criterion were then used to fit the optimal model.

**Results:**

This study included 399 patients. Most had grade 1 or 2 (86.2%) tumors, originating mostly in the small intestine (43.9%) and pancreas (41.6%). Prevalence of malnutrition and sarcopenia was 61.9 and 15%, respectively. The predictive model had an external accuracy of 65.3 and 71.2% for malnutrition and sarcopenia, respectively. The stronger predictive factors for malnutrition were poor tumor differentiation (odds ratio [OR]: 4.4; 95% confidence interval [CI]: 1.2–22), nausea/vomiting (OR: 3.4; 95% CI: 1.4–9.3), having difficulties for a long walk (OR: 3; 95% CI: 1.8–5) and diabetes (OR: 2.2; 95% CI: 1.3–3.9). Sarcopenia was correlated with having troubles in activities of daily living (OR: 19.7; 95% CI: 3.1–390.9), or difficulties for a long walk (OR: 6.1; 95% CI: 2.6–15.5).

**Conclusion:**

A nomogram based on these models was developed. We generated a diagnostic tool for malnutrition and sarcopenia in patients with GEP-NENs to identify high-risk cases.

## Introduction

Neuroendocrine neoplasms (NENs) are rare malignancies, accounting for up to 8.61 cases per 100,000 individuals (1) primarily originating from neuroendocrine cells in the gastroenteropancreatic (GEP) tract. The most prevalent primary tumor sites include the foregut, midgut, and hindgut, followed by the pancreas.

GEP-NENs can significantly impact patient nutritional status because of the following factors: (1) anatomical alterations in the GEP tract caused by the tumor, (2) disruption of normal organ function, potentially leading to metabolic disorders such as diabetes, and (3) secretion of bioactive substances (peptides and hormones) by tumor cells, which may cause different hypersecretion disorders, such as carcinoid syndrome (2–7). Furthermore, surgical and therapeutic management of GEP-NENs may disrupt GEP function, with potential dietary and nutritional implications (8, 9). Previous studies have reported malnutrition rates ranging from 30 to 70% in patients with GEP-NETs, depending on the cancer type, with higher rates in advanced stages (8, 10–14).

Nutritional status is relevant because it may impair the efficacy of anticancer treatments and undermine the patient’s quality of life, potentially leading to a worse prognosis (15). Consequently, there is a need to develop accessible, easy-to-use screening tools that facilitate the rapid and cost-effective identification of cancer patients at risk of malnutrition, enabling early targeted nutritional assessment and intervention by specialized nutritionists or endocrinologists in such cases. Existing tools for nutritional risk assessment, such as PREvención con DIeta MEDiterranea (PREDIMED), Malnutrition Universal Screening Tool (MUST), and Subjective Global Assessment (SGA) tests, were designed to detect malnutrition in a broader general population. However, they do not specifically address the needs of cancer patients, and more precisely, those with GEP-NENs, a population at high risk of malnutrition. Therefore, these screening tools may lack sensitivity in GEP-NENs (16–18); in fact, previous reports showed a sensitivity of 46 and 41% for MUST and SGA, respectively (19).

The NUTRIGETNE study included 399 evaluable patients, representing the largest cohort of advanced GEP-NENs with a comprehensive nutritional and functional assessment reported to date (19). The prevalence of malnutrition and sarcopenia were 61.9 and 15%, respectively (19). In this study, we exploited the full set of clinical characteristics collected in this study to develop a nomogram to aid the assessment of the risk of malnutrition and sarcopenia in patients with GEP-NENs.

## Methods

### Study design

The NUTRIGETNE study (NCT04986085) was an observational, cross-sectional, prospective, epidemiologic, multicenter study led by the Grupo Español de Tumores Neuroendocrinos y Endocrinos (GETNE) and including patients across 17 reference hospitals in Spain. The primary aim was to describe the nutritional status of patients with advanced GEP-NENs receiving anticancer treatment in accordance with the standard practices in Spain. After describing the population of patients included in the NUTRIGETNE study, the creation of a model capable of predicting the risk of malnutrition and sarcopenia was proposed as an exploratory objective. The creation of a tool to aid the oncologists in determining malnutrition in patients was also proposed as an exploratory objective. The trial was initially approved by the Ethics Committee of the Hospital Universitario y Politécnico la Fe de Valencia (ref: 2020-704-1) in March 2021 and by the corresponding participant centers. The study was conducted in compliance with the Declaration of Helsinki and applicable local regulatory laws. Informed consent was obtained from all patients prior to their inclusion.

### Patient characteristics

The NUTRIGETNE study included patients with a histologically confirmed diagnosis of GEP-NEN with unresectable locally advanced or metastatic stage, aged between 18 and 80 years, who were treated at inclusion following standard clinical practice. Treatment modalities included somatostatin analogs (SSA), targeted therapies, chemotherapy (CT), radionuclides (PRRT), or other standard systemic or locoregional therapies. Patients were systematically screened and consecutively included during their visits to the respective health centers for outpatient care or hospitalization. The decision to prescribe these anticancer treatments was made independently of patient participation in this study. Pregnant women, patients undergoing palliative treatment, those in the terminal stage, and those lacking histological confirmation of the disease were excluded.

### Study assessments and endpoints

The study comprised a single clinical visit during which patients underwent a comprehensive physical examination conducted by a nutritionist or specialized physician. The visit included anthropometry and routine laboratory blood analysis. Adverse events (AEs) reported during the visit were coded and graded according to the National Cancer Institute Common Terminology Criteria for Adverse Events. Additionally, patients were interviewed and asked to complete two quality of life questionnaires: the European Organization for Research and Treatment of Cancer QLQ-C30 and the specific module for NETs, QLQ-GINET. In total, the study comprised 478 variables.

Malnutrition was defined in accordance with the Global Leadership Initiative on Malnutrition (GLIM) criteria (20). The phenotypic criteria used were unintended weight loss (yes/no), low BMI (yes/no), and low muscle mass (yes/no). The etiological criteria used were reduced intake or assimilation (yes/no) and inflammatory burden due to underlying disease (yes/no). Sarcopenia was defined, according to the European Working Group on Sarcopenia in Older People 2 (EWGSOP2) criteria, as low muscle mass in bioelectrical impedance coinciding with low muscle performance as assessed by handgrip strength (21).

### Data processing

The dataset was thoroughly reviewed to identify missing values. Patients were randomized in a ratio of 4:1 into training and validation sets. Variables with a maximum missing data percentage of 5% and a *p*-value < 0.1 in the training set were selected to construct the models. The *p*-values used were corrected for the false discovery rate. Final predictive models for malnutrition and sarcopenia were constructed using 222 and 212 variables, respectively. Patients with missing data for the selected variables were excluded from the analysis to ensure the robustness of the findings and final calculator.

### LASSO variable selection strategy

Random oversampling by patient duplication was applied to the training sample to achieve balanced groups regarding the dependent variables, malnutrition, and sarcopenia. All selected variables were used to predict malnutrition and sarcopenia. The model was adjusted for sex, age, and body mass index (BMI). Bootstrapping with 100 iterations was used to identify the most parsimonious predictive models using a 10-fold cross-validated least absolute shrinkage and selection operator (LASSO) model with the optimal value of lambda (λ*). Variables that remained in the models for >75% of the time were selected as predictors.

### Best logistic regression to determine the risk of malnutrition and sarcopenia

Logistic regression analysis was used to determine whether the selected variables were significantly associated with malnutrition and sarcopenia. The most suitable predictors were used to fit the best possible logistic regression models. The accuracy of the models was evaluated in the training and validation sets for internal and external validation. A predictive score was generated for each patient based on the final model. Calibration receiver operating characteristic (ROC) curves were used to determine the cutoff points for the risk of malnutrition or sarcopenia.

### Statistical analysis

Continuous variables were summarized using descriptive statistics. Normality was assessed using the Saphiro-Wilk test, and parametric and nonparametric tests were used as appropriate. All statistical tests were two-tailed, and statistical significance was set at *p* < 0.05. Multiple testing adjustments were not performed. All statistical analyses were performed using R software (version 4.3.2 [2023], Boston, MA, US).

## Results

### Patient characteristics

From July 2021 to July 2023, 399 patients were included in the NUTRIGETNE study, with a median age of 62 years (range: 22–83). Most patients were men (57.1%) and had grade 1 or 2 tumors (86.2%). Most tumors originated in the small intestine (43.9%) and pancreas (41.6%). Patient characteristics have been described elsewhere (19). The prevalence of malnutrition and sarcopenia were 61.9 and 15%, respectively (19). Malnutrition and sarcopenia were diagnosed concurrently in 60 patients (25.6%), being all sarcopenic patients malnourished as well (Supplementary Figure 1). At the moment of determination of nutritional status, 54.4% of patients received or were receiving the first line, 23.1% the second, and 22.6% the third or subsequents. These treatments consisted mainly in SSA (86%), PRRT (27%), tyrosine kinase inhibitors (27%), or CT (20%). The predictive models for malnutrition and sarcopenia departed from 365 and 362 patients, respectively, because some patients had missing information on several outcomes and were excluded from the analysis. The patient characteristics of these subgroups are shown in Table 1.

**Table 1:** Baseline patient characteristics in the whole population used for modeling the prediction tool.

Characteristic	Malnutrition (GLIM)*		*p*-value	Sarcopenia (EWGSOP)*		*p*-value
	Yes *N* = 230	No *N* = 135		Yes *N* = 55	No *N* = 307	
Median age (range); years	63 (22–83)	60 (30–81)	0.185^1^	72 (53–81)	61 (22–83)	<0.001^1^
Sex; *n* (%)						
Male	123 (53.5)	84 (62.2)	0.126^2^	28 (50.9)	178 (58.0)	0.376^2^
Female	107 (46.5)	51 (37.8)		27 (49.1)	129 (42.0)	
Race; *n* (%)						
Caucasian	217 (94.3)	130 (96.3)	0.558^3^	54 (98.2)	291 (94.8)	0.361^3^
Hispanic	8 (3.5)	4 (3.0)		0 (0)	11 (3.6)	
African	5 (2.2)	1 (0.7)		1 (1.8)	5 (1.6)	
ECOG-PS; *n* (%)						
Score 0	110 (47.8)	81 (60.0)	<0.001^1^	22 (40.0)	171 (55.7)	<0.001^1^
Score 1	79 (34.3)	40 (29.6)		22 (40.0)	90 (29.3)	
Score ≥ 2	19 (8.3)	0 (0)		10 (18.2)	8 (2.6)	
Unknown	22 (9.6)	14 (10.4)		1 (1.8)	38 (12.4)	
Tumor grade WHO; *n* (%)^a^						
Grade 1	90 (39.1)	48 (35.6)	0.592^4^	22 (40.0)	111 (36.2)	0.214^4^
Grade 2	110 (47.8)	68 (50.4)		30 (54.5)	149 (48.5)	
Grade 3	28 (12.2)	17 (12.6)		3 (5.5)	43 (14.0)	
Unknown	2 (0.9)	2 (1.5)		0 (0)	4 (1.3)	
Differentiation; *n* (%)						
NET	209 (90.9)	130 (96.3)	0.059^1^	44 (80.0)	283 (92.2)	0.038^1^
NEC	21 (9.1)	5 (3.7)		8 (14.5)	18 (5.9)	
Unknown	0 (0)	0 (0)		3 (5.5)	6 (2)	
Functionality; *n* (%)						
Yes	55 (23.9)	34 (25.2)	0.802^1^	8 (14.5)	82 (26.7)	0.062^1^
No	172 (74.8)	100 (74.1)		47 (85.5)	220 (71.7)	
Unknown	3 (1.3)	1 (0.7)		0 (0)	5 (1.6)	
Primary tumor location, *n* (%)						
Small intestine	97 (42.2)	63 (46.7)	0.340^3^	19 (34.5)	140 (45.6)	0.516^3^
Pancreas	97 (42.2)	56 (41.5)		26 (47.3)	128 (41.7)	
Colorectal	13 (5.7)	3 (2.2)		4 (7.3)	12 (3.9)	
Gastric	6 (2.6)	1 (0.7)		3 (5.5)	5 (1.6)	
Other/unknown	17 (7.4)	12 (8.9)		3 (5.5)	22 (7.2)	
Metastasis at inclusion, *n* (%)						
0**	10 (4.3)	5 (3.7)	0.730^4^	1 (1.8)	14 (4.6)	0.600^4^
1	134 (58.3)	73 (54.1)		31 (56.4)	174 (56.7)	
≥2	86 (37.4)	57 (42.2)		23 (41.8)	119 (38.8)	
Previous lines; *n* (%)						
1	123 (53.5)	73 (54.1)	0.521^4^	22 (40.0)	172 (56.0)	0.084^4^
2	51 (22.2)	36 (26.7)		18 (32.7)	66 (21.5)	
>2	56 (24.3)	26 (19.3)		15 (27.3)	69 (22.5)	

ECOG PS, Eastern Cooperative Oncology Group Performance Status; EWGSOP, European Working Group on Sarcopenia in Older People; GLIM, Global Leadership Initiative on Malnutrition; NEC, neuroendocrine carcinoma; NET, neuroendocrine tumor; PRRT, peptide receptor radionuclides; SSA, somatostatin analogs; TKIs, tyrosine kinase inhibitors; WHO, World Health Organization.

*Only patients included in the predictive model. ** Patients with unresectable locally advanced disease.

^1^Fisher’s exact test.

^2^Mann-Whitney test.

^3^Pearson chi-squared test.

^4^Linear-by-linear association test.

Most patient characteristics at baseline were balanced. Malnourished patients reported a significantly worse baseline performance status (ECOG ≥2: 8.3% vs. 0% for malnourished and non-malnourished patients, respectively; *p* < 0.001). Patients with sarcopenia were older (median age 72 vs. 61 years; *p* < 0.001), had worse performance status (ECOG 2: 18.2% vs. 2.6%; *p* = 0.001), and had a higher number of NECs (14.5% vs. 5.9%; *p* = 0.038). Patient characteristics in the training and validation sets are shown in Supplementary Tables 1, 2.

### Malnutrition predictive model

The partial-likelihood deviance was lowest when 12 variables (Supplementary Figure 2), including age, sex, and BMI (confounding variables), were incorporated. The model was pruned following the variable selection method to obtain the most parsimonious model that included the most related variables alongside confounders; a total of eight final variables were retained. The following variables showed the strongest statistically significant correlations with malnutrition: presence of diabetes, poorly differentiated histological tumor grade, nausea and vomiting symptoms, reporting having any trouble taking a long walk, and reporting weight loss problems quite a bit or more frequently (Table 2). Patients with NECs had 4.4-fold increased probability of malnutrition, suggesting a relevant role for tumor histology in tumor-induced nutritional worsening.

**Table 2:** Regression coefficients of the most powerful factors identified by the LASSO regression analysis for the malnutrition model.

Characteristic	Comparison	ODDs Ratio (95% CI)	*p*-value
*Age	old vs. young (per year)	1.20 (0.99–1.03)	0.454
*Sex	Male vs. Female	1.20 (0.73–1.97)	0.472
*BMI	high vs. low (per kg/m^2^)	0.86 (0.80–0.92)	<0.001
Diabetes	present vs. absent	2.25 (1.31–3.89)	0.003
Histological differentiation	NEC vs. NET	4.45 (1.22–22.03)	0.038
Nausea and vomiting	present vs. absent	3.38 (1.38–9.26)	0.011
QlQ2. Do you have any trouble taking a long walk?	Quite a bit/Very Much/A little vs. Not at all	3.01 (1.83–5.02)	<0.001
QlQ45. Weight loss problem?	Quite a bit/Very Much vs. Not at all/A little	2.39 (1.11–5.40)	0.030

BMI, body mass index; CI, confidence interval; NEC, neuroendocrine carcinoma; NET, neuroendocrine tumor; QlQ, quality of life questionnaire.

*Confounding factors included in the final model.

**Figure 1:**
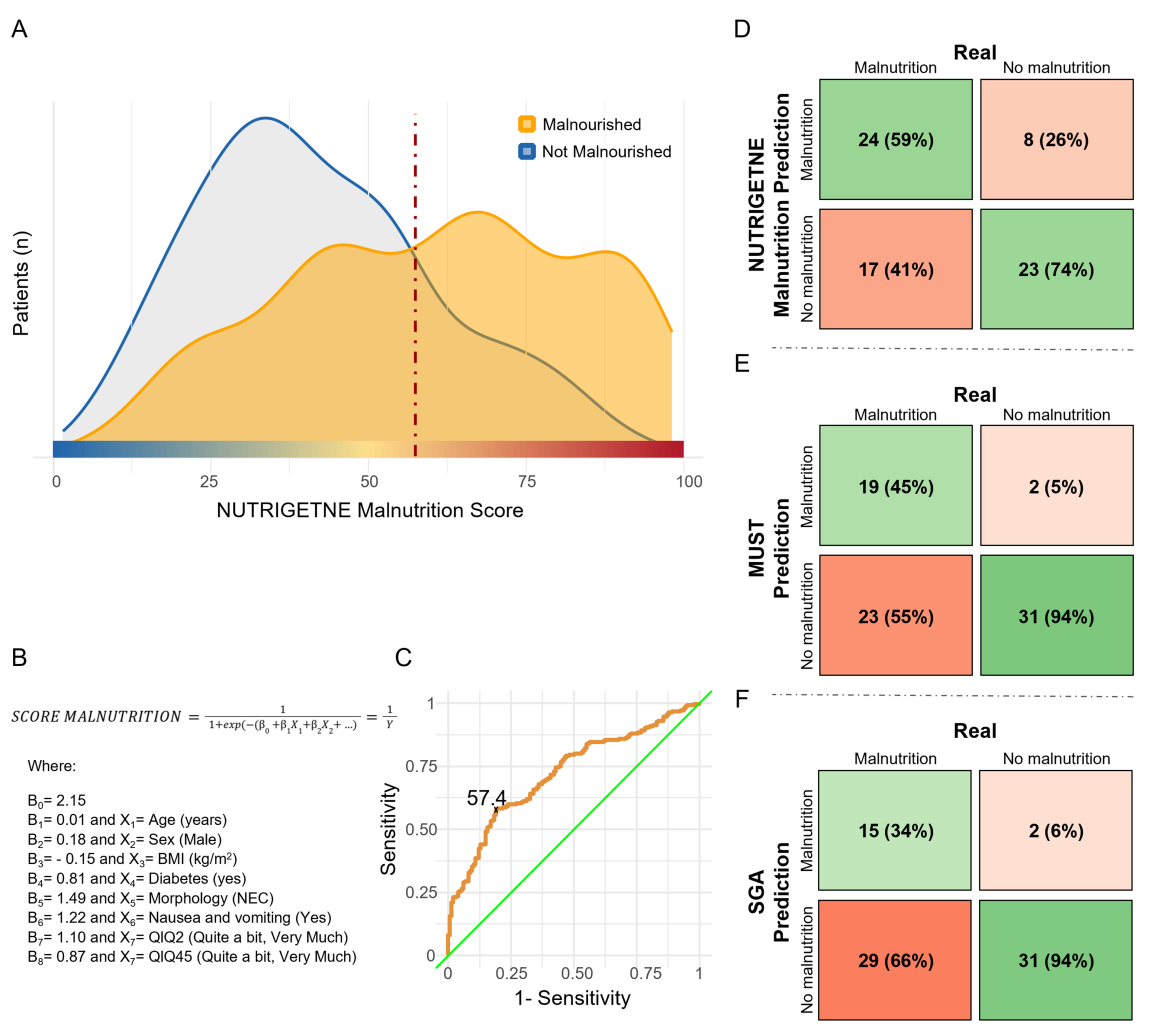
Malnutrition predictive model and score. **(A)** Histogram with patient distribution according to the NUTRIGETNE malnutrition score, depicted on the *X*-axis. The population was subdivided into patients with malnutrition according to the GLIM criteria (orange) and patients with no malnutrition (gray area under the blue curve) to show the distribution of each group through the NUTRIGETNE malnutrition risk score. The red dashed line represents the risk score cutoff value in the model. **(B)** Formula used to obtain the NUTRIGETNE Malnutrition score. **(C)** ROC curve in the full dataset showing the best score cut off point. The area under the curve was 0.720 (95% CI: 0.670–0.771). **(D–F)** Confusion matrix showing the prediction of the NUTRIGETNE malnutrition prediction tool **(D)**, MUST test **(E)**, and SGA test **(F)** in the validation set. Correlation between the prediction and the real GLIM malnutrition status is shown. Greener colors indicate a higher rate of correct assignments, and reddish colors indicate a higher rate of incorrectly assigned patients.

The malnutrition model was constructed using the variables with strongest association with the condition, and a risk score was generated. The distribution of patients in the validation set, as assessed by the NUTRIGETNE malnutrition score, demonstrated a good correlation (Figures 1A,B). Subsequently, the ROC curve of the prediction model was generated. The area under the curve (AUC) in the validation cohort was 0.720 (95% CI: 0.670–0.771), indicating an acceptable predictive value. A risk score of 57.4 was established as the cutoff value with the highest Youden index (Figure 1C). Based on this cutoff risk score, patients were categorized into two groups: high-risk and low-risk of malnutrition.

The predictive model performance was tested against the validation subset, revealing an external accuracy (i.e., how closely the model’s predictions matched the actual patient status) of 65.3% and sensitivity (i.e., ability to correctly identify malnourished cases) of 58.5% (Figure 1D). The performances of MUST and SGA tests were also assessed in the validation set, reaching accuracies and sensitivities of 66.7 and 45.2%, respectively (Figure 1E), and 59.7 and 34.1%, respectively (Figure 1F).

### Sarcopenia predictive model

The same variable selection method used for malnutrition was employed. The optimal model for sarcopenia incorporated six variables with the strongest associations, including confounding variables. Sarcopenia was significantly correlated in the final model with three questions from quality of life (QoL) questionnaires: experiencing difficulties taking a long walk quite a bit or more often, requiring assistance for daily activities quite a bit or more often, and rating overall QoL below a score of 2 (Table 3). Notably, patients who reported a need for assistance while eating, dressing, washing themselves, or using the toilet frequently exhibited a 19.7-fold increased probability of sarcopenia.

**Table 3:** Regression coefficients of the most powerful factors identified by the LASSO regression analysis for the sarcopenia model.

Characteristic	Comparison	ODDs Ratio (95% CI)	*p*-value
*Age	old vs. young (per year)	1.15 (1.12–1.18)	<0.001
*Sex	Male vs. Female	1.30 (0.81–2.09)	0.278
*BMI	high vs. low (per kg/m^2^)	0.89 (0.84–0.94)	<0.001
QlQ2. Do you have any trouble taking a long walk?	Quite a bit/Very Much vs. Not at all/A little	6.07 (2.62–15.53)	<0.001
QlQ30. How would you rate your overall quality of life during the past week?	Score >2 vs. ≤2	0.25 (0.10–0.59)	0.002
QlQ5. Do you need help with eating, dressing, washing yourself or using the toilet?	Quite a bit/Very Much vs. Not at all/A little	19.70 (3.06–390.85)	0.008

BMI, body mass index; CI, confidence interval; QlQ, quality of life questionnaire.

*Confounding factors included in the final model.

**Figure 2:**
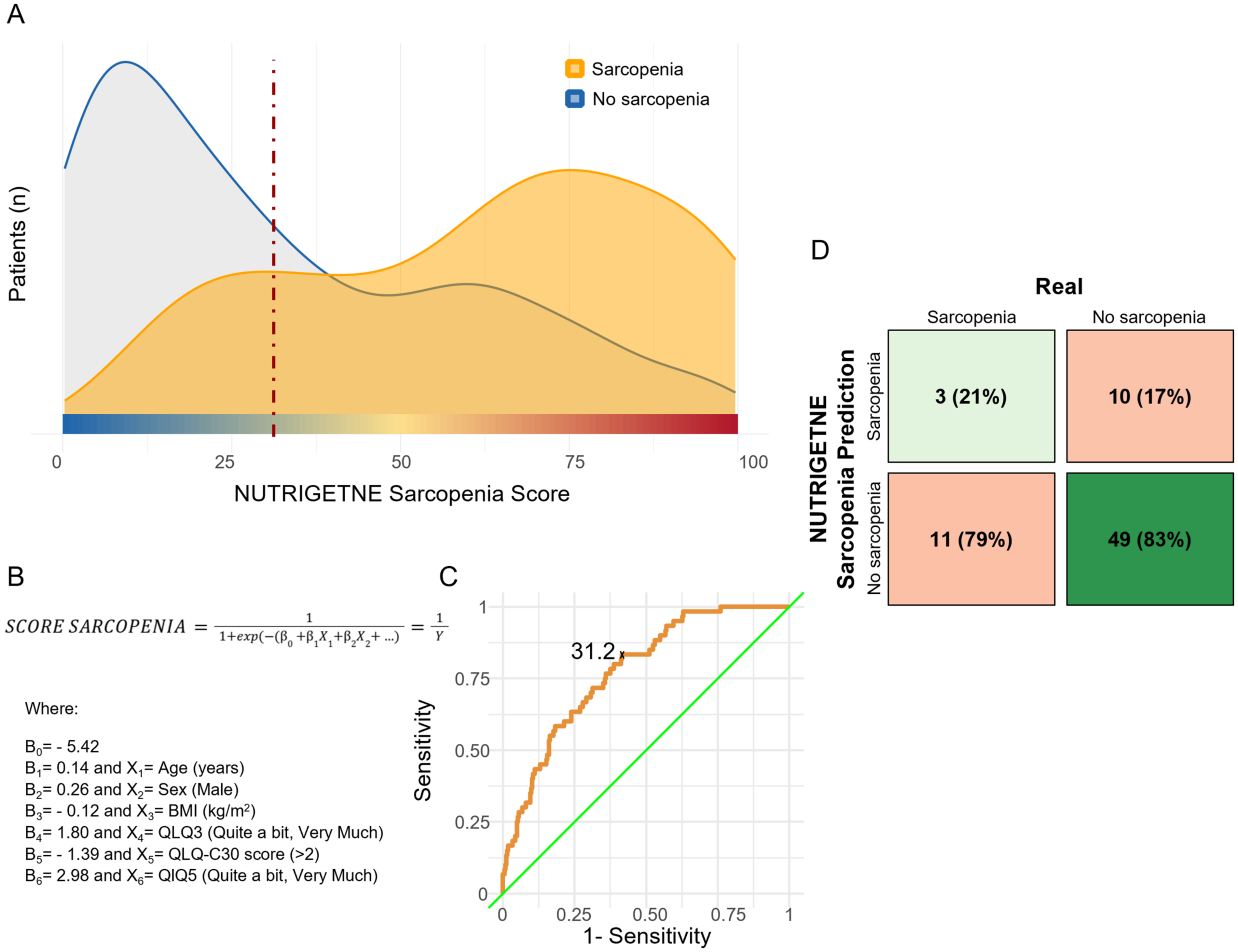
Sarcopenia predictive model and score. **(A)** Histogram with patient distribution according to the NUTRIGETNE sarcopenia score, depicted on the *X*-axis. The population was subdivided into patients with sarcopenia according to the EWGSOP criteria (orange) and patients with no sarcopenia (gray area under the blue curve) to show the distribution of each group through the NUTRIGETNE sarcopenia risk score. The red dashed line represents the risk score cutoff value in the model. **(B)** Formula used to obtain the NUTRIGETNE sarcopenia score. **(C)** ROC curve in the full dataset showing the best score cut off point. The area under the curve was 0.777 (95% CI: 0.719–0.833). **(D)** Coincidence matrix showing the prediction of the NUTRIGETNE sarcopenia prediction tool in the validation set. The correlation between the prediction and the real EWGSOP sarcopenia status is shown. Greener colors indicate higher rate of correct assignments and reddish colors indicate higher rate of incorrectly assigned patients.

The distribution of patients according to the risk score of the sarcopenia predictive model showed a strong correlation (Figures 2A,B). The AUC in the validation cohort was 0.777 (95% CI: 0.719–0.833), indicating acceptable predictive value. A risk score of 31.2 was established as the cutoff value with the highest Youden index (Figure 2C). The performance of the sarcopenia predictive model was evaluated, demonstrating an accuracy and sensitivity in the validation set of 71.2 and 21.4%, respectively (Figure 2D).

### Final nomogram models for prediction of malnutrition and sarcopenia

**Figure 3:**
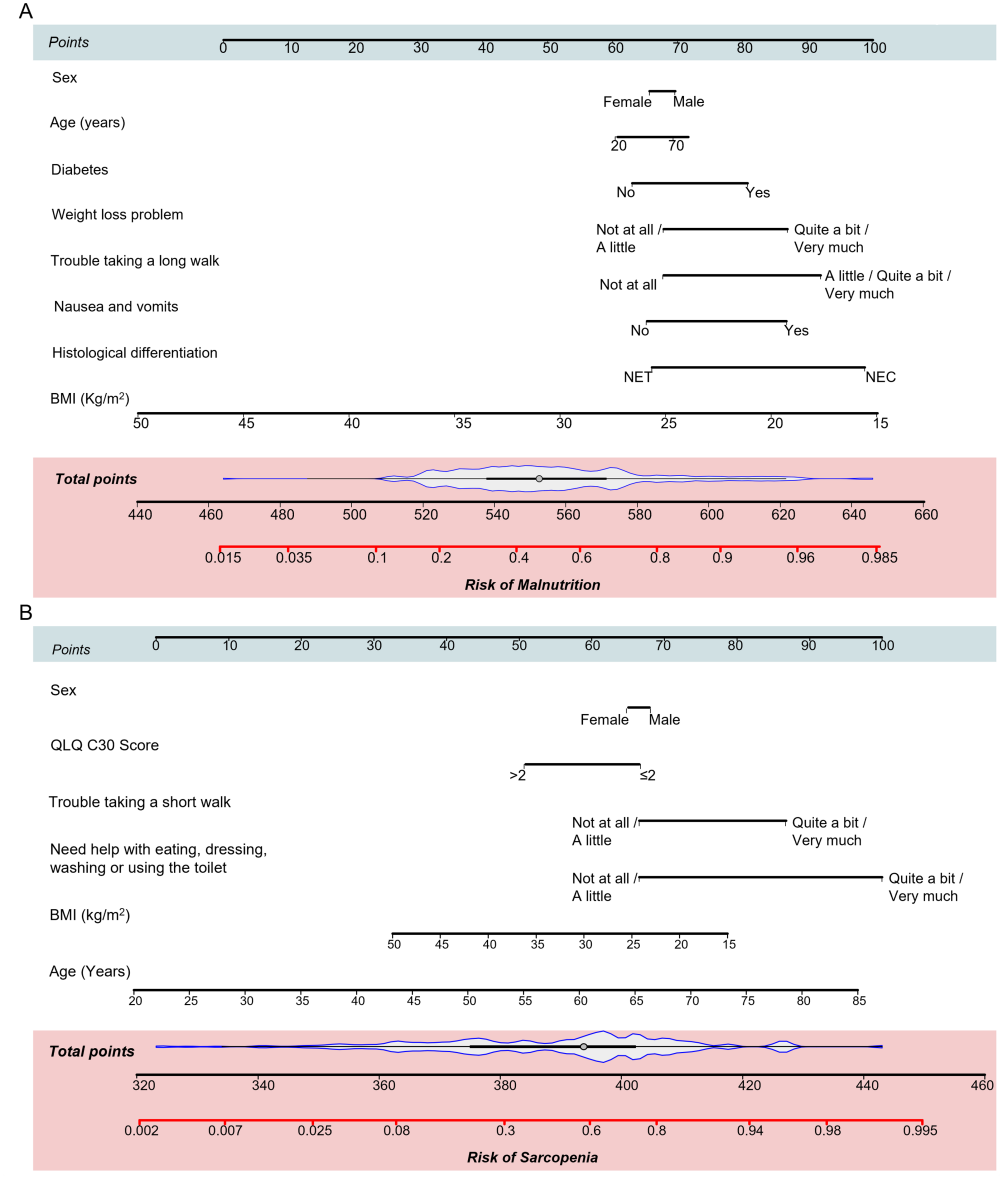
Nomogram for predicting malnutrition according to the GLIM criteria **(A)** and sarcopenia according to the EWGSOP criteria **(B)**. To utilize the model to forecast the probability of malnutrition and sarcopenia in patients with GEP-NENs, a risk score for each variable (line above over the blue area) was first derived from the patient’s clinical information. The risk may be calculated for each variable using a and a plumb line through that point. The sum of the risk score (black line below over the red area) for each variable is correlated with the probability of malnutrition and sarcopenia (red line below over the red area). The violin plots show the distribution of patients in the study population across the scores.

A nomogram based on these predictive models was developed to assess the risk of malnutrition and sarcopenia in patients with GEP-NENs (Figures 3A,B and Supplementary Figures 3, 4). The malnutrition model assigned a substantial weight to BMI, histological differentiation, and QoL Accordingly, changes in these variables resulted in greater risk score differences. The sarcopenia model assigned greater weight to age, BMI, and QoL.

## Discussion

The interest in the nutritional status of patients with cancer has been growing in the last decades, as numerous studies demonstrated high rates of malnutrition and sarcopenia and their potential impact on prognosis (8, 10–13, 15, 19, 22). Given the prevalence and implications of malnutrition, systematic monitoring throughout the disease course should be recommended, although this presents challenges. Less than one-third of patients undergo regular nutritional assessments (22, 23). In fact, a systematic complete evaluation of all patients would be impractical because of time and financial constraints. Furthermore, there is no consensus on the optimal screening tools to identify patients in greater need of nutritional assessment.

The NUTRIGETNE study represents the largest population of GEP-NENs with comprehensive nutritional and functional assessments. The study comprises a representative real-world population and relevant datasets to identify potential predictive factors of malnutrition in GEP-NENs, accounting for a total of 478 variables. This is the first report dedicated to the creation of predictive screening tools specifically for GEP-NENs.

The NUTRIGETNE predictive models for malnutrition and sarcopenia may provide a rapid, cost-effective, and highly sensitive tool. Both models require only basic clinical determinations and a brief patient interview to assess the risk of malnutrition and sarcopenia. The malnutrition model appeared to demonstrate greater adaptability to the GEP-NENs population and exhibited higher sensitivity than the currently employed methods, MUST and SGA, in our population (sensitivity of 58.5% vs. 45.2% vs. 34.1%, respectively). Our model shows the potential to predict malnutrition in approximately six out of 10 patients with the condition and could serve as a valuable tool for initial screening. The nomograms based on these predictive models may provide a straightforward tool to aid in determining malnutrition during outpatient visits with oncologists. However, the sensitivity, specially for sarcopenia, should be improved to be more useful to identify malnourished and sarcopenic patients. Functionality has been proposed to influence the nutritional status of patients with NENs (24). However, as previously reported (19), our models did not find functionality, tumor grade and primary tumor locations as main covariates of malnutrition and sarcopenia.

Our predictive model showed that diabetes increased the risk of malnutrition by 2.25-fold. In our population and in previous studies, a high prevalence of diabetes among patients with GEP-NENs have been reported (19, 25, 26). Diabetes has been suggested as a potential risk factor for the development and prognosis of GEP-NENs (25, 26), but the underlying effects remain unclear. Insulin resistance, compensatory insulinemia, or vitamin D deficiency, have been associated with nutritional status (26–29). Moreover, recent literature has proposed “diabetic sarcopenia” as a distinct comorbidity of diabetes, characterized by muscle atrophy and functional impairment (30).

Histological differentiation has emerged as a major determinant in our predictive model of malnutrition and poorly differentiated carcinomas (NECs) associated with a 4.45-fold increase in the risk of malnutrition. This association has been corroborated in previous studies (8, 10–13, 22). Based on these findings, patients with NECs and concurrent diabetes might be at an elevated risk of malnutrition, which may warrant a comprehensive and continuous nutritional assessment.

Symptoms of nausea and vomiting, which have proven to be useful indicators of malnutrition, are prevalent among malnourished patients (13). These symptoms, often induced by chemotherapy, SSAs, or other anticancer treatments, may contribute to reduced food intake, potentially exacerbating malnutrition (2, 24).

Classical and demographic characteristics including BMI, sex, and age were included to adjust the models. The consistent weight of BMI in the final models confirms its relevance. Our results suggest that BMI remains a key factor in malnutrition and sarcopenia, whereas age also plays a pivotal role in sarcopenia. Therefore, it is essential to include these variables in routine nutritional assessments alongside QoL and newly identified relevant factors. In addition, incorporating BMI-adjusted percentiles for muscle mass and function in the future may improve the assessment of nutritional and functional status, support the precise identification of at-risk individuals, and inform tailored interventions (31).

Interestingly, several parameters related to the patient’s perception of their health status and ability to perform activities of daily living emerged as independent predictive indicators of malnutrition and sarcopenia. Critical questions in the quality of life questionnaires were identified. Specifically, the most relevant items were as follows: the ability to take a long walk or perform activities of daily living, reporting weight loss problems, requiring help for daily activities, and rating the overall quality of life ≤2. These findings may contribute to a more targeted assessment of patient-reported outcomes with minimal discomfort and rapid risk determination during consultations. Our results highlight the importance of actively listening to patients’ perceptions of their disease and health status, as this may be more strongly correlated with their nutritional status than traditional measures, such as BMI.

Recently, the assessment of albumin-myosteatosis gauge has been proposed as a surrogate of malnutrition (32). Taking into account that the baseline albumin was different between patients with and without malnutrition, it would be interesting to recollect data regarding myosteatosis.

The main limitation of the predictive models might lie in their currently low accuracy and the potential for misdiagnosing a number of patients with malnutrition or sarcopenia. Broader external validation and further refinement are necessary. Nevertheless, our models achieved higher sensitivity than MUST or SGA within our study population, which is expected to behave similarly to the real-world population of GEP-NENs. The model could be strengthened through its systematic application in the real world as part of comprehensive nutritional assessments. Owing to the study design and the significant patient heterogeneity in rare diseases like NECs and NENs, another limitation arises from these characteristics, potentially introducing bias due to the small sample size of certain subgroups. The high number of variables initially analyzed could generate overfitting or increase the *α* error. However, several correction measures were applied, such as a false discovery rate correction to *p*-values or constructing the final models with a low number of variables, with eight for malnutrition and six for sarcopenia.

In conclusion, the NUTRIGETNE Score is positioned as an accessible, rapid, and cost-effective screening tool designed to facilitate risk-based nutritional assessment for patients with GEP-NENs. The NUTRIGETNE Score is suggested for incorporation as part of routine monitoring for patients with GEP-NENs by oncologists to guide referrals to endocrinologists/nutritionists for consultation and interventions.

## Data Availability

The data is available from the corresponding author upon reasonable request (equivalent purposes to those for which the patients grant their consent to use the data). Data will be provided anonymously, with no identifiable data.

## Group member of NUTRIGETNE group

Paula Jiménez-Fonseca (Medical Oncology; Hospital Universitario Central de Asturias, Instituto de Investigación Sanitaria del Principado de Asturias (ISPA), Oviedo, Spain), Teresa Alonso-Gordoa, Alvaro Ruiz Grandos, and Noemi Brox (Medical Oncology; Hospital Universitario Ramón y Cajal, Madrid, Spain), Ana Isabel Castro Pais, Navia Duyos Mato, and José Manuel Cabezas Agrícola (Endocrinology and Nutrition Department, Complejo Hospitalario Universitario de Santiago, Santiago de Compostela (A Coruña), Spain), Marta Diéguez, Lucía Díaz, Pilar Monge, and Carmen Gándara (Endocrinology and Nutrition Department, Hospital Universitario de Cabueñes, Gijón, Spain), Anna Casteras (Endocrinology Department, Vall d´Hebron University Hospital), Alejandro García-Álvarez (Medical Oncology Department, Vall d´Hebron University Hospital), Andrea Micó Garcia (Endocrinology and Nutrition department) University Hospital La Fe (Valencia).
