# Supplementary material for: A prediction tool for malnutrition and sarcopenia in patients with gastroenteropancreatic neuroendocrine neoplasms: results from NUTRIGETNE (GETNE-S2109) study
Source: Front Nutr. 2026 May 26;13:1789458. doi: 10.3389/fnut.2026.1789458 (PMC13246423; doi:10.3389/fnut.2026.1789458)
Supplement: Supplementary file 1 [file Table_1.DOCX]

**Supplementary Table 1. Baseline patient characteristics in the training and validation population used for the malnutrition model.** *Abbreviations: ECOG PS, Eastern Cooperative Oncology Group Performance Status; GLIM, Global Leadership Initiative on Malnutrition; NEC, neuroendocrine carcinoma; NET, neuroendocrine tumor; PRRT, peptide receptor radionuclides; SSA, somatostatin analogs; TKIs, tyrosine kinase inhibitors; WHO, World Health Organization.*

| **Characteristic** | **Training** | | **p-value** | **Validation** | | **p-value** |
| --- | --- | --- | --- | --- | --- | --- |
| **Malnutrition (GLIM)** | **Yes**  **N=189** | **No**  **N= 104** |  | **Yes**  **N= 41** | **No**  **N= 31** |  |
| Median age (range); years | 63 (22-83) | 62 (31-81) | 0.328 ^1^ | 63 (22-81) | 55 (30-72) | 0.002 ^1^ |
| Sex; n (%) | | | | | | |
| Male | 101 (53.4) | 60 (57.7) | 0.540 ^2^ | 22 (53.7) | 24 (77.4) | 0.049 ^2^ |
| Female | 88 (46.6) | 44 (42.3) |  | 19 (46.3) | 7 (22.6) |  |
| Race; n (%) | | | | | | |
| Caucasian | 179 (94.7) | 100 (96.2) | 0.757 ^3^ | 38 (92.7) | 30 (96.8) | 0.637 ^3^ |
| Hispanic | 6 (3.2) | 3 (2.9) |  | 2 (4.9) | 1 (3.2) |  |
| African | 4 (2.1) | 1 (1.0) |  | 1 (2.4) | 0 (0) |  |
| ECOG-PS; n (%) | | | | | | |
| Score 0 | 92 (48.7) | 61 (58.7) | 0.002 ^1^ | 18 (43.9) | 20 (64.5) | 0.024 ^1^ |
| Score 1 | 63 (33.3) | 32 (30.8) |  | 16 (39.0) | 8 (25.8) |  |
| Score ≥ 2 | 15 (7.9) | 0 (0.0) |  | 4 (9.8) | 0 (0) |  |
| Unknown | 19 (10.1) | 11 (10.6) |  | 3 (7.3) | 3 (9.7) |  |
| Tumor grade WHO; n (%) **^a^** | | | | | | |
| Grade 1 | 78 (41.3) | 37 (35.6) | 0.505 ^4^ | 12 (29.3) | 11 (35.5) | 0.768 ^4^ |
| Grade 2 | 87 (46.0) | 53 (51.0) |  | 23 (56.1) | 15 (48.4) |  |
| Grade 3 | 22 (11.6) | 12 (11.5) |  | 6 (14.6) | 5 (16.1) |  |
| Unknown | 2 (1.1) | 2 (1.9) |  | 0 (0) | 0 (0) |  |
| Differentiation; n (%) | | | | | | |
| NET | 172 (91.0) | 102 (98.1) | 0.023 ^1^ | 37 (90.2) | 28 (90.3) | 1.000 ^1^ |
| NEC | 17 (9.0) | 2 (1.9) |  | 4 (9.8) | 3 (9.7) |  |
| Functionality; n (%) | | | | | | |
| Yes | 43 (22.8) | 30 (28.8) | 0.262 ^1^ | 12 (29.3) | 4 (12.9) | 0.152 ^1^ |
| No | 143 (75.7) | 73 (70.2) |  | 29 (70.7) | 27 (87.1) |  |
| Unknown | 3 (1.6) | 1 (1) |  | 0 (0) | 0 (0) |  |
| Primary tumor location, n (%) | | | | | | |
| Small intestine | 78 (41.3) | 53 (51) | 0.171 ^3^ | 19 (46.3) | 10 (32.3) | 0.479 ^3^ |
| Pancreas | 80 (42.3) | 40 (38.5) |  | 17 (41.5) | 16 (51.6) |  |
| Colorectal | 10 (5.3) | 1 (1) |  | 3 (7.3) | 3 (9.7) |  |
| Gastric | 4 (2.1) | 1 (1) |  | 2 (4.9) | 0 (0) |  |
| Other / unknown | 17 (9) | 9 (8.7) |  | 0 (0) | 2 (6.5) |  |
| Metastasis at inclusion, n (%) | | | | | | |
| 0 | 8 (4.2) | 3 (2.9) | 0.734 ^4^ | 2 (4.9) | 2 (6.5) | 0.989 ^4^ |
| 1 | 112 (59.3) | 60 (57.7) |  | 22 (53.7) | 13 (41.9) |  |
| ≥ 2 | 69 (36.5) | 41 (39.4) |  | 17 (41.5) | 16 (51.6) |  |
| Previous lines; n (%) | | | | | | |
| 1 | 102 (54.0) | 54 (51.9) | 0.723 ^4^ | 21 (51.2) | 19 (61.3) | 0.479 ^4^ |
| 2 | 42 (22.2) | 31 (29.8) |  | 9 (22.0) | 5 (16.1) |  |
| > 2 | 45 (23.8) | 19 (18.3) |  | 11 (26.8) | 7 (22.6) |  |

**Only patients included in the predictive model*

1. *Fisher's exact test*
2. *Mann-Whitney test*
3. *Pearson chi-squared test*
4. *Linear-by-linear association test*
