# Supplementary material for: A prediction tool for malnutrition and sarcopenia in patients with gastroenteropancreatic neuroendocrine neoplasms: results from NUTRIGETNE (GETNE-S2109) study
Source: Front Nutr. 2026 May 26;13:1789458. doi: 10.3389/fnut.2026.1789458 (PMC13246423; doi:10.3389/fnut.2026.1789458)
Supplement: Supplementary file 2 [file Table_2.DOCX]

**Supplementary Table 2. Baseline patient characteristics in the whole population used for the sarcopenia model.** *Abbreviations: ECOG PS, Eastern Cooperative Oncology Group Performance Status; EWGSOP, European Working Group on Sarcopenia in Older People; NEC, neuroendocrine carcinoma; NET, neuroendocrine tumor; PRRT, peptide receptor radionuclides; SSA, somatostatin analogs; TKIs, tyrosine kinase inhibitors; WHO, World Health Organization.*

| **Characteristic** | **Training** | | **p-value** | **Validation** | | **p-value** |
| --- | --- | --- | --- | --- | --- | --- |
| **Sarcopenia (EWGSOP)** | **Yes**  **N=41** | **No**  **N= 248** |  | **Yes**  **N= 14** | **No**  **N= 59** |  |
| Median age (range); years | 73 (53-80) | 61 (22-83) | < 0.001 ^1^ | 68 (58-81) | 57 (22-80) | 0.001 ^1^ |
| Sex; n (%) | | | | | | |
| Male | 21 (51.2) | 138 (55.6) | 0.615 ^2^ | 7 (50.0) | 40 (67.8) | 0.230 ^2^ |
| Female | 20 (48.8) | 110 (44.4) |  | 7 (50.0) | 19 (32.2) |  |
| Race; n (%) | | | | | | |
| Caucasian | 41 (100.0) | 234 (94.4) | 0.296 ^3^ | 13 (92.9) | 57 (96.6) | 0.095 ^3^ |
| Hispanic | 0 (0) | 9 (3.6) |  | 0 (0) | 2 (3.4) |  |
| African | 0 (0) | 5 (2.0) |  | 1 (7.1) | 0 (0) |  |
| ECOG-PS; n (%) | | | | | | |
| Score 0 | 15 (36.6) | 139 (56.0) | < 0.001 ^1^ | 7 (50.0) | 32 (54.2) | 0.581 ^1^ |
| Score 1 | 16 (39.0) | 73 (29.4) |  | 6 (42.9) | 17 (28.8) |  |
| Score ≥ 2 | 9 (22.0) | 5 (2.0) |  | 1 (7.1) | 3 (5.1) |  |
| Unknown | 1 (2.4) | 31 (12.5) |  | 0 (0) | 7 (11.9) |  |
| Tumor grade WHO; n (%) **^a^** | | | | | | |
| Grade 1 | 18 (43.9) | 93 (37.5) | 0.199 ^4^ | 4 (28.6) | 18 (30.5) | 0.688 ^4^ |
| Grade 2 | 21 (51.2) | 118 (47.6) |  | 9 (64.3) | 31 (52.5) |  |
| Grade 3 | 2 (4.9) | 33 (13.3) |  | 1 (7.1) | 10 (16.9) |  |
| Unknown | 0 (0) | 4 (1.6) |  | 0 (0) | 0 (0) |  |
| Differentiation; n (%) | | | | | | |
| NET | 33 (80.5) | 229 (92.3) | 0.033 ^1^ | 11 (78.6) | 54 (91.5) | 0.602 ^1^ |
| NEC | 6 (14.6) | 13 (5.2) |  | 2 (14.3) | 5 (8.5) |  |
| Unknown | 2 (4.9) | 6 (2.4) |  | 1 (7.1) | 0 (0) |  |
| Functionality; n (%) | | | | | | |
| Yes | 7 (17.1) | 66 (26.6) | 0.245 ^1^ | 1 (7.1) | 16 (27.1) | 0.165 ^1^ |
| No | 34 (82.9) | 177 (71.4) |  | 13 (92.9) | 43 (72.9) |  |
| Unknown | 0 (0) | 5 (2.0) |  | 0 (0) | 0 (0) |  |
| Primary tumor location, n (%) | | | | | | |
| Small intestine | 14 (34.1) | 117 (47.2) | 0.505 ^3^ | 5 (35.7) | 26 (44.1) | 0.761 ^3^ |
| Pancreas | 19 (46.3) | 102 (41.1) |  | 7 (50.0) | 26 (44.1) |  |
| Colorectal | 3 (7.3) | 8 (3.2) |  | 1 (7.1) | 4 (6.8) |  |
| Gastric | 2 (4.9) | 4 (1.6) |  | 1 (7.1) | 1 (1.7) |  |
| Other / unknown | 3 (7.3) | 17 (6.9) |  | 0 (0) | 2 (3.4) |  |
| Metastasis at inclusion, n (%) | | | | | | |
| 0 | 1 (2.4) | 10 (4.0) | 0.725 ^4^ | 0 (0) | 4 (6.8) | 0.721 ^4^ |
| 1 | 24 (58.5) | 146 (58.9) |  | 7 (50.0) | 28 (47.5) |  |
| ≥ 2 | 16 (39.0) | 92 (37.1) |  | 7 (50.0) | 27 (5.4) |  |
| Previous lines; n (%) | | | | | | |
| 1 | 15 (36.6) | 138 (55.6) | 0.079 ^4^ | 7 (50.0) | 34 (57.6) | 0.674 ^4^ |
| 2 | 15 (36.6) | 56 (22.6) |  | 3 (21.4) | 10 (16.9) |  |
| > 2 | 11 (26.8) | 54 (21.8) |  | 4 (28.6) | 15 (25.4) |  |

**Only patients included in the predictive model*

1. *Fisher's exact test*
2. *Mann-Whitney test*
3. *Pearson chi-squared test*
4. *Linear-by-linear association test*
