# Supplementary material for: A prediction tool for malnutrition and sarcopenia in patients with gastroenteropancreatic neuroendocrine neoplasms: results from NUTRIGETNE (GETNE-S2109) study
Source: Front Nutr. 2026 May 26;13:1789458. doi: 10.3389/fnut.2026.1789458 (PMC13246423; doi:10.3389/fnut.2026.1789458)
Supplement: Supplementary file 3 [file Table_3.DOCX]

**Supplementary table 3. Quality of life questionnaire glossary.**

| **QUESTIONS** |
| --- |
| Question 1: Do you have any trouble doing strenuous activities, like carrying a heavy shopping bag or a suitcase? |
| Question 2: Do you have any trouble taking a long walk? |
| Question 3: Do you have any trouble taking a short walk outside of the house? |
| Question 4: Do you need to stay in bed or a chair during the day? |
| Question 5: Do you need help with eating, dressing, washing yourself or using the toilet? |
| Question 6: Were you limited in doing either your work or other daily activities? |
| Question 7: Were you limited in pursuing your hobbies or other leisure time activities? |
| Question 8: Were you short of breath? |
| Question 9: Have you had pain? |
| Question 10: Did you need to rest? |
| Question 11: Have you had trouble sleeping? |
| Question 12: Have you felt weak? |
| Question 13: Have you lacked appetite? |
| Question 14: Have you felt nauseated? |
| Question 15: Have you vomited? |
| Question 16: Have you been constipated? |
| Question 17: Have you had diarrhea? |
| Question 18: Were you tired? |
| Question 19: Did pain interfere with your daily activities? |
| Question 20: Have you had difficulty in concentrating on things, like reading a newspaper or watching television? |
| Question 21: Did you feel tense? |
| Question 22: Did you worry? |
| Question 23: Did you feel irritable? |
| Question 24: Did you feel depressed? |
| Question 25: Have you had difficulty remembering things? |
| Question 26: Has your physical condition or medical treatment interfered with your family life? |
| Question 27: Has your physical condition or medical treatment interfered with your social activities? |
| Question 28: Has your physical condition or medical treatment caused you financial difficulties? |
| Question 29: How would you rate your overall health during the past week? |
| Question 30: How would you rate your overall quality of life during the past week? |
| Question 31: Hot flushes |
| Question 32: Looked flushed red |
| Question 33: Night sweats |
| Question 34: Abdominal discomfort |
| Question 35: Bloated feeling in abdomen |
| Question 36: Problem passing gas/flatulence |
| Question 37: Acid indigestion/heartburn |
| Question 38: Difficulties eating |
| Question 39: Side effects from treatments |
| Question 40: Problem from repeated injections |
| Question 41: Worried about metastasis |
| Question 42: Concerned about disruption of home life |
| Question 43: Worried of health in future |
| Question 44: Distress for those close |
| Question 45: Weight loss problem |
| Question 46: Weight gain problem |
| Question 47: Worried about test results |
| Question 48: Aches/ pain in muscle and bones |
| Question 49: Limitations to travel |
| Question 50: Misinformation about disease and treatment |
| Question 51: Worsen of sex life |
