# Supplementary material for: A prediction tool for malnutrition and sarcopenia in patients with gastroenteropancreatic neuroendocrine neoplasms: results from NUTRIGETNE (GETNE-S2109) study
Source: Front Nutr. 2026 May 26;13:1789458. doi: 10.3389/fnut.2026.1789458 (PMC13246423; doi:10.3389/fnut.2026.1789458)
Supplement: Supplementary file 4 [file Image_1.PDF]

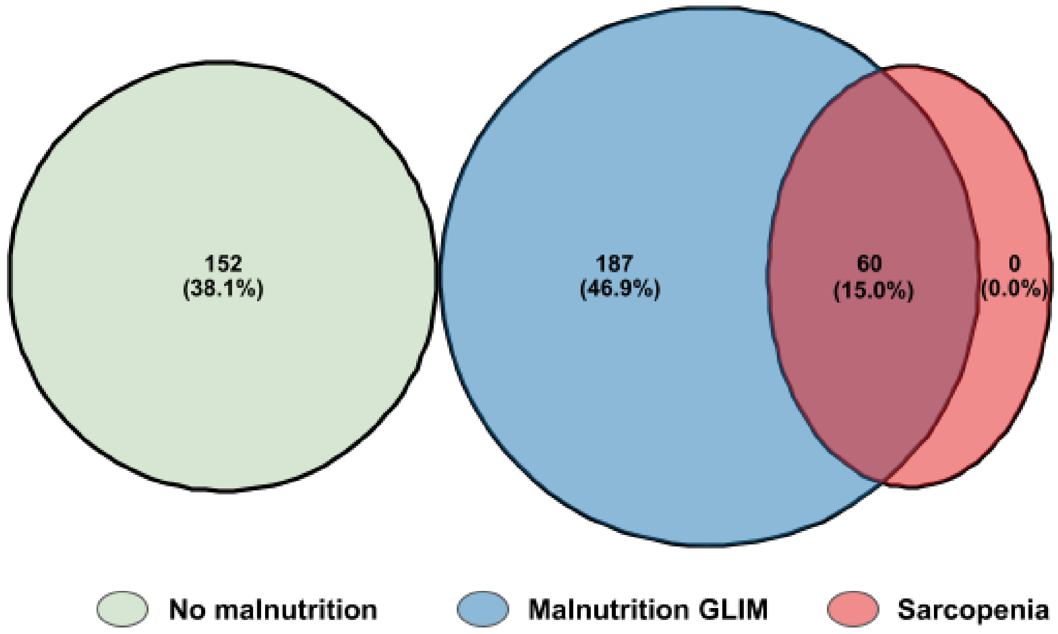

Supplementary figure 1. Venn diagram showing the concordance of malnutrition according to GLIM criteria (blue) and sarcopenia according to EWGSOP criteria (red). Patients without malnutrition according to GLIM or sarcopenia are shown in green.
