# Supplementary material for: A prediction tool for malnutrition and sarcopenia in patients with gastroenteropancreatic neuroendocrine neoplasms: results from NUTRIGETNE (GETNE-S2109) study
Source: Front Nutr. 2026 May 26;13:1789458. doi: 10.3389/fnut.2026.1789458 (PMC13246423; doi:10.3389/fnut.2026.1789458)
Supplement: Supplementary file 5 [file Image_2.PDF]

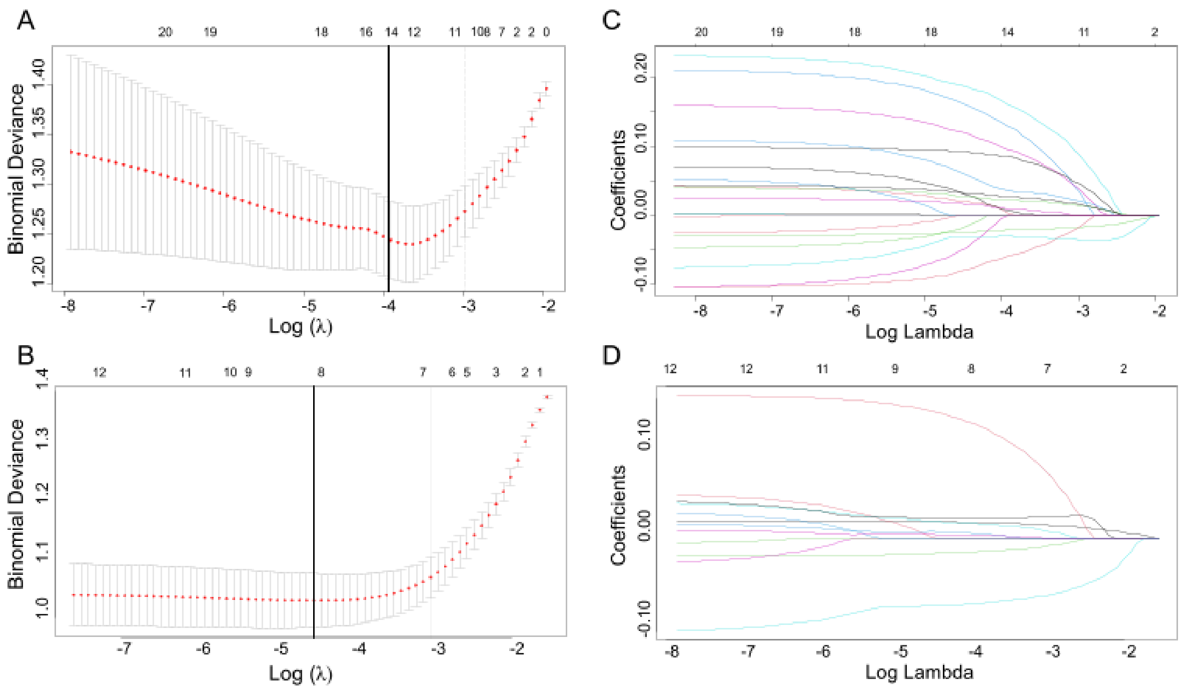

Supplementary figure 2. Identification of the influencing factors by LASSO regression for malnutrition (A) and sarcopenia (B). The LASSO regression using an optimal alpha identified the following 11 most powerful predictors of malnutrition: diabetes, food intake, handgrip, histological differentiation, nausea and vomiting, need of rest, having trouble taking a long walk, physical condition or medical treatment interfering with social activities, overall quality of life during the past week, having difficulties eating, and having weight loss problems. The LASSO regression identified the following 4 most powerful predictors of sarcopenia: having any trouble doing strenuous activities, like carrying a heavy shopping bag or a suitcase, having any trouble taking a short walk outside of the house, overall quality of life during the past week, and requiring help with eating, dressing, washing or using the toilet. Plot C-D) shows the coefficients change as the value of lambda varies when all predictors were included in the LASSO regression models of malnutrition and sarcopenia, respectively.
