# Supplementary material for: A prediction tool for malnutrition and sarcopenia in patients with gastroenteropancreatic neuroendocrine neoplasms: results from NUTRIGETNE (GETNE-S2109) study
Source: Front Nutr. 2026 May 26;13:1789458. doi: 10.3389/fnut.2026.1789458 (PMC13246423; doi:10.3389/fnut.2026.1789458)
Supplement: Supplementary file 6 [file Image_3.PDF]

Variable	Category	Score
Sex	Male	70
	Female	66
Age (years)	20	60
	45	65
	70	69
Diabetes	Yes	81
	No	63
Weight loss problem	Not at all / A little	68
	Quite a bit / Very much	87
Trouble taking long walk	Not at all	68
	A Little / Quite a bit / Very much	92
Nausea and vomits	Yes	93
	No	65
Histological differentiation	NET	66
	NEC	99
BMI (Kg/m^2)	15	100
	30	52
	45	3

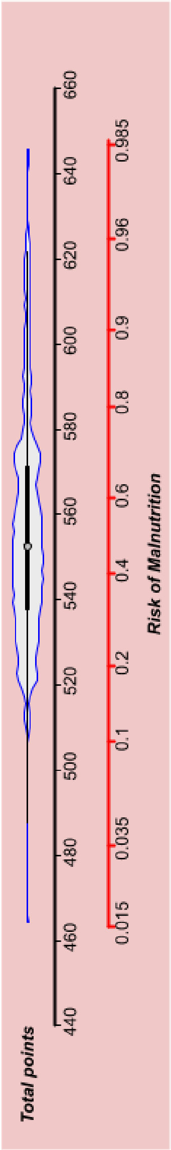

Supplementary figure 3. Simplified categoric nomogram to predict the risk of malnutrition according to GLIM criteria.
