# Supplementary material for: A prediction tool for malnutrition and sarcopenia in patients with gastroenteropancreatic neuroendocrine neoplasms: results from NUTRIGETNE (GETNE-S2109) study
Source: Front Nutr. 2026 May 26;13:1789458. doi: 10.3389/fnut.2026.1789458 (PMC13246423; doi:10.3389/fnut.2026.1789458)
Supplement: Supplementary file 7 [file Image_4.PDF]

Variable	Category	Score
Sex	Male	68
	Female	65
QLQ C30 Score	>2	51
	<=2	67
Trouble taking a short walk	Not at all / A little	67
	Quite a bit / Very much	87
Need help with eating, dressing, washing or using toilet	Not at all / A little	67
	Quite a bit / Very much	100
BMI (Kg/m^2)	15	79
	30	59
	45	39
Age (years)	25	5
	50	43
	75	81

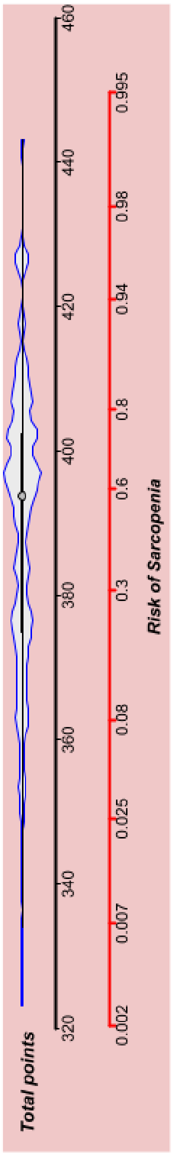

Supplementary figure 4. Simplified categoric nomogram to predict the risk of sarcopenia according to EWGSOP criteria.
